# Nailfold video-capillaroscopy in the study of cardiovascular disease: a systematic review

**DOI:** 10.1097/MBP.0000000000000624

**Published:** 2022-10-20

**Authors:** Matthew W.S. Lim, Dellaneira Setjiadi, Stephen J.H. Dobbin, Ninian N. Lang, Christian Delles, Paul J. Connelly

**Affiliations:** School of Cardiovascular and Metabolic Health, University of Glasgow, Glasgow, UK

**Keywords:** capillaries, cardiovascular diseases, laser-Doppler, flowmetry, microscopic angioscopy, thermography

## Abstract

Nailfold video-capillaroscopy (NVC) is an inexpensive method of assessing microcirculation. We reviewed the literature to assess whether changes to the nailfold capillaries exist in patients with cardiovascular disease (CVD).

**Methods:**

We searched *PubMed, Scopus* and *Cochrane Library* databases for original research articles relating to the use of noninvasive microvascular assessment in patients with CVD. Methodological quality was assessed with the ‘Quality Assessment Tool for Observational Cohort and Cross-sectional Studies.’ The results obtained from NVC were analysed qualitatively and compared with other forms of microvascular assessment.

**Results:**

In total 2759 articles were screened, of which 22 studies involving 562 patients (~40% women) with CVD were included. Mean age ranged between 3.7–68.4 years (cases) and 4.0–58.0 years (controls). Reduced capillary density and increased capillary dimensions were seen in patients with pulmonary arterial hypertension (PAH). Among patients with systemic sclerosis, advanced scleroderma patterns can be used to identify patients with or at risk of developing PAH. Functional nailfold changes precede structural changes in patients with hypertension. However, the studies were heterogeneous in the diagnosis of disease and the measurement of nailfold parameters. Most studies did not exclude conditions with altered nailfold features, and only one study performed a power calculation. Furthermore, abnormal nailfold findings are present in patients without systemic disease.

**Conclusions:**

Structural and functional changes to the nailfold are a feature of established CVD and precede the development of PAH. However, heterogeneity in measurement and abnormal findings in healthy participants limit their use in the wider population.

## Introduction

Resistance arteries are responsible for the perfusion of organs and tissues, as well as for maintaining peripheral vascular resistance [1]. Small resistance arteries (lumen diameter <150 µm), along with arterioles, capillaries and venules form the microcirculation [2]. Given the integral role of the microcirculation in normal physiology, microvascular dysfunction in cardiovascular disease (CVD) should be evaluated to further our understanding of cardiovascular pathophysiology, which may lead to the identification of novel therapeutic targets.

Several means of assessing the microvasculature exist. Tissue perfusion may be assessed using a laser beam (laser-Doppler flowmetry and laser speckle contrast imaging) [3,4] or infrared radiation (thermography) [5]. Peripheral arterial tonometry has been used to predict adverse cardiovascular events [6]. However being noninvasive, peripheral arterial tonometry assesses both macrovascular and microvascular function.

Video-capillaroscopy is the visualisation of the capillaries [7,8] often performed in the nailfolds. The horizontally oriented capillaries in the nailfolds enable the assessment of the entire capillary loop, including dimensions and morphology [8]. Nailfold video-capillaroscopy (NVC) has achieved considerable success in rheumatological conditions, with abnormal nailfold findings used in the diagnostic criteria of systemic sclerosis [9] and monitoring of disease progression [10].

CVD is common in patients with connective tissue disease and represents a major cause of death. In people lacking traditional cardiovascular risk factors, atherosclerotic CVD was approximately three times as prevalent compared with control populations [11]. However, the evidence supporting the use of NVC as a surrogate for CVD in patients without connective tissue disease is limited. We aimed to review the literature on the use of NVC in patients with CVD. We hypothesise that functional and structural changes to the nailfold capillaries are more common in patients with CVD.

## Methods

### Literature search

This review adhered to the Preferred Reporting Items for Systematic Reviews and Meta-Analyses statement [12]. We included randomised controlled trials and observational studies using NVC in patients of all ages with cardiovascular disease (as defined by the National Library of Medicine [13]) and a suitable comparator group. Articles written in foreign languages and secondary research articles were excluded. Studies involving patients with the systemic disease were eligible provided that both groups were comparable apart from the CVD of interest.

A search was performed in *PubMed, Scopus* and *Cochrane Library* databases for articles published until 14 January 2022. The following Medical Subject Headings terms were used: ‘Microscopic Angioscopy’ OR ‘Thermography’ OR ‘Laser-Doppler Flowmetry’ AND ‘Cardiovascular Diseases’. The results were entered into EndNote X9. The protocol has been registered on PROSPERO (CRD42021286490).

### Data extraction and quality assessment

Two independent reviewers (M.L. and D.S.) performed a literature search, data extraction and quality assessment. Disagreements were resolved by consensus or the involvement of a third reviewer (P.C.). Titles and abstracts were screened to identify eligible articles, which were retrieved and read in full. Reference lists from the selected articles were examined to identify additional articles.

Data extracted included the name of the first author, publication year, study design, sample size, NVC measurement (site and nailfold parameters) and key findings. The results were entered into Microsoft Excel spreadsheets and analysed qualitatively. A meta-analysis was not performed as the studies were heterogenous in the diagnosis of CVD and measurement of nailfold parameters.

To assess methodological quality, we used the National Institute of Health Quality Assessment Tool for Observational Cohort and Cross-Sectional Studies [14]. This tool consisted of 14 questions. Using the answers compiled, the studies were rated as good, fair or poor. Inter-rater agreement was assessed using Cohen’s kappa [15]. A kappa value between 0.4 and 0.6 is considered moderate, 0.6 and 0.8 is substantial and values greater than 0.8 as almost perfect agreement. All statistical analyses were performed in IBM SPSS statistics (version 28.0).

## Results

### Study characteristics

**Fig. 1:**
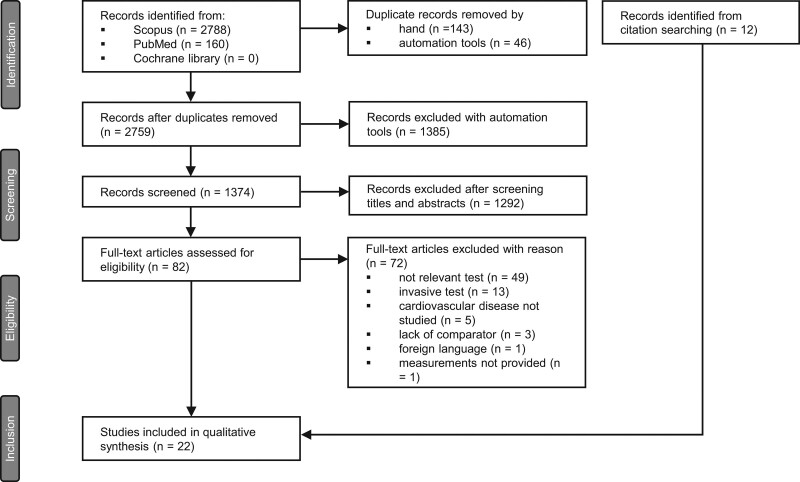
Study selection.

The initial search identified 2948 records (Fig. 1). After excluding duplicate records, foreign language articles, grey literature and animal studies, we selected 1374 original research articles for the title and abstract screening, of which 82 full texts were assessed for eligibility. In total 72 articles were excluded due to video-capillaroscopy measurements performed at sites other than the nailfolds [16,17], the use of invasive microvascular assessments [18], CVD not being studied [19] or other reasons (Supplementary Table S1, Supplemental digital content, http://links.lww.com/BPMJ/A182). The remaining 10 articles and an additional 12 articles identified from citation searching were included in this systematic review.

The studies were published from 1997 to 2021 and included a total of 562 patients with CVD (~40% female). Mean ages ranged from 3.7 to 68.4 years (cases) and 4.0 to 58.0 years (controls) [20–22]. Among the included studies, two were cohort studies with a mean follow-up period ranging from 25 to 27 months [23,24]. The remaining studies were cross-sectional studies.

### Parameters

**Fig. 2:**
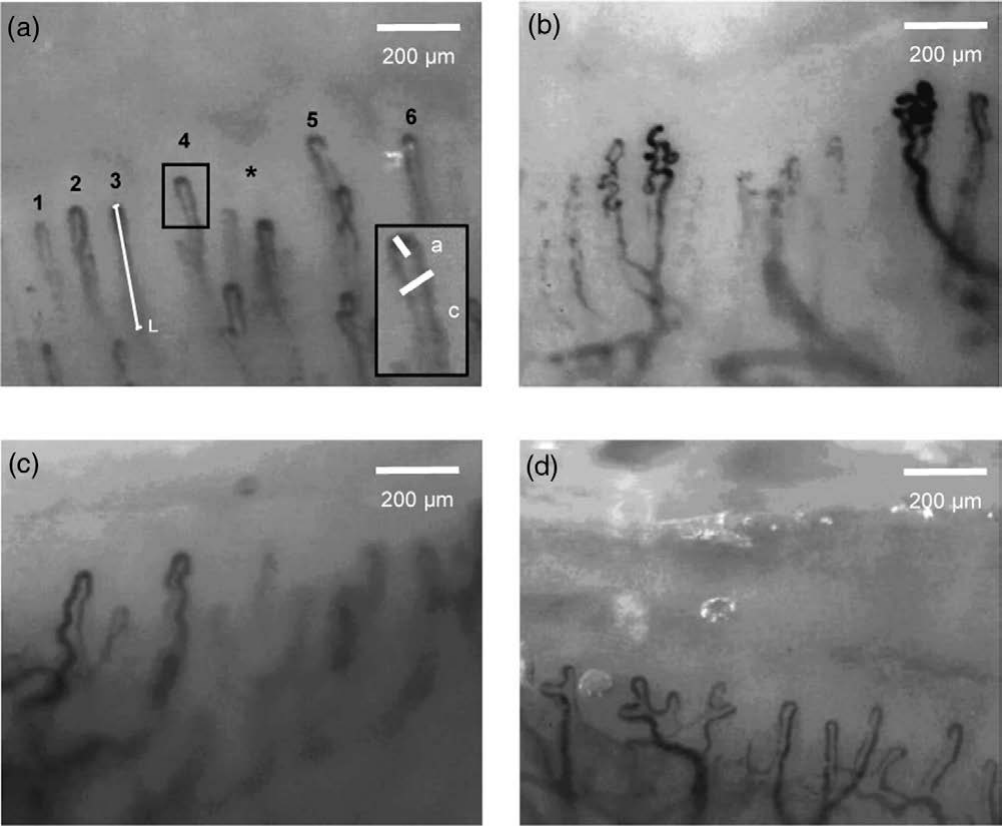
(a) Normal capillaries are uniform, thin and hairpin shaped. Capillary density is calculated as the number of distal capillary loops (numbered) per mm. An avascular area (asterisk) is described as the loss of two or more distal capillaries. Capillary length (L) is measured from the base to the apex of the capillary. Insert shows the capillary dimensions: capillary width (c) and apical diameter (a). The capillary width is the external diameter of the capillary at its widest point. This is the sum of the arterial limb, venous limb, and the internal diameter of the capillaries. Blood flows from the left to right, as such the arterial limb is positioned to the right of the venous limb. The apical diameter is measured at the apex and represents the widest point of the capillary loop. (b) Bushy capillaries show small buddings. (c) Tortuous capillaries are limbs that curl but do no cross. (d) Ramified capillaries have branches. Images were taken at the Clinical Research Facility, Queen Elizabeth University Hospital (Glasgow, UK).

Capillary density is commonly defined as the number of distal capillary loops per mm (Fig. 2a). The total capillary loops in 1 mm^2^ may also be measured [25–28]. An avascular area is described as the loss of two or more distal capillaries. At baseline, only the continuously perfused capillaries are visualised. To visualise the intermittently perfused capillaries, nailfold measurements are taken after arterial occlusion. The recruited capillaries are known as the functional reserve capillaries [25]. In addition, venous occlusion may be used to visualise the maximal capillary density (the sum of perfused and nonperfused capillaries) [25]. All measurements were taken at baseline unless otherwise specified.

Capillary dimension is another important parameter (Fig. 2a). Capillary width is the external diameter of the capillary at its widest point. This is the sum of the arterial limb, venous limb and internal diameter of the capillary. Apical diameter is the width of the capillary loop measured at the apex, which represents the widest point of the capillary loop. Additionally, capillary length is measured from the apex to the base of the capillary where the loop ceases to be visible [29].

The normal capillary is hairpin-shaped with a venous limb wider than the arterial limb (Fig. 2a). Morphological features may be present in patients with CVD (Fig. 2b–d). Capillaries with small buddings are known as bushy capillaries. Tortuous capillaries are curled limbs that do not cross. In addition, capillaries that branch are known as ramified capillaries.

Using frame-to-frame analysis software, the flow of individual red blood cells can be visualised. From this recording, the speed of travel (red cell velocity) is calculated [26,30]. Although a useful marker of capillary function, this measurement is technically difficult and not routinely performed [26,30].

### Pulmonary hypertension

Nailfold changes were present across the spectrum of pre-capillary pulmonary hypertension (Table 1). Three main subtypes of pulmonary arterial hypertension (PAH) were investigated: idiopathic, PAH-associated with congenital heart disease and PAH-associated with connective tissue disease.

**Table 1:** Nailfold findings in patients with pulmonary hypertension

First author	*N* a	Subtype	CD	LD	Abnormal morphology
Arvanitaki *et al*., [31]	14 (30)	Idiopathic	**↓**	**↑**	Ramifications
Corrado *et al*., [32]	21(20)		**↓**	**↑**	None
Hofstee *et al*., [33]	20 (21)		**↓**	**↔**	
Greidinger *et al*., [34]^b^	37 (13)				None
Arvanitaki *et al*., [35]	17 (17)	Eisenmenger syndrome	**↓**	**↑**	Avascular areas, haemorrhage, thrombosis, and ramifications
Arvanitaki *et al*., [21]	13 (14)	Systemic sclerosis	**↓**	**↑**	Scleroderma patterns
Corrado *et al*., [32]	19 (20)		**↓**	**↑**	Bushy
Hofstee *et al*., [33]	21 (19)		**↓**	**↔**	
Voilliot *et al*., [23]^b^	11 (29)				Scleroderma patterns
Riccieri *et al*., [36]	12 (12)		**↓**		Scleroderma patterns and avascular areas
Ohtsuka *et al*., [37]	10 (34)				Scleroderma patterns
Marino *et al*., [38]^b^	5 (39)				None
Ong *et al*., [22]^b^	8 (12)	Limited scleroderma	**↓**	**↔**	
Arvanitaki *et al*., [31]	14 (30)	Chronic thromboembolic	**↓**	**↑**	Thrombosis and ramifications

CD, capillary density; LD, loop dimensions; N, sample size.

a Sample size of patients with the disease of interest and the number of controls in brackets.

b diagnosis confirmed with echocardiography; ↑ increased; ↓ decreased; ↔ preserved.

In four studies involving patients with idiopathic PAH [31–34], the diagnosis was confirmed with right heart catheterisation in three studies [31–33], whereas the remaining study included patients diagnosed by echocardiography [34]. When compared with healthy controls, patients with idiopathic PAH had reduced capillary density [31–33] and increased capillary dimensions [31,32].

In a study involving 14 patients with idiopathic PAH and 30 healthy controls, Arvanitaki *et al.* [31]. reported reduced capillary density (8.4 vs. 9.7 loops/mm; *P* < 0.001), increased capillary width (35.1 vs. 29.5 μm; *P* < 0.01) and apical diameter (15.7 vs 11.5 μm; *P* < 0.001). In addition, patients with idiopathic PAH had an increased number of avascular areas (*P* < 0.01) but the proportion of patients with at least one avascular area was similar. Two adjacent 1 mm fields were observed in all fingers excluding the thumbs. The results agree with a previous study which showed reduced capillary density and increased apical diameter [32].

In addition, Hosftee *et al.* [33] demonstrated that capillary density was negatively correlated with mean pulmonary artery pressure (mPAP; *r* = −0.67; *P*  = 0.001). However, capillary dimensions did not differ. The study design may explain the difference: capillary dimensions were calculated from the widest capillaries. Whereas in previous studies, measurements were taken from all the visible capillaries [31,32]. This suggests that nailfold capillaries are wider on average but enlarged capillaries may be present in patients without systemic disease.

PAH is frequent in patients with congenital heart disease. In Eisenmenger syndrome, a left-to-right shunt leads to increased pulmonary vascular resistance and pulmonary artery pressure [35]. Arvanitaki *et al.* reported reduced capillary density (8.8 vs. 9.9 loops/mm; *P*  = 0.004) and increased capillary width (35.6 vs. 27.5 μm; *P*  = 0.001) in patients with Eisenmenger syndrome [35]. Morphological changes were nonspecific but were correlated with *N*-terminal pro-brain natriuretic peptide (*r* = 0.52; *P*  = 0.03).

Connective tissue disease, another major cause of PAH, has been investigated in eight studies. The diagnosis was confirmed by right heart catheterisation in five studies [21,32,33,36,37], whereas the three remaining studies also included patients diagnosed with echocardiography [22,23,38]. Among patients with systemic sclerosis, those with PAH have increased capillary width [21,32] and reduced capillary density [21,32,33]. Capillary density was negatively correlated with mPAP (*r* = −0.58; *P* = 0.039) [33]. Of the three studies, Hofstee *et al.* did not find any differences in capillary dimensions [33]. As discussed earlier, the use of the widest capillaries instead of all visible capillaries may explain the differences.

Abnormal morphology is a distinguishing feature of PAH secondary to connective tissue disease. Among patients with systemic sclerosis, those with PAH had advanced (active and late) scleroderma patterns [21,36]. Scleroderma grades correlated with mPAP (*r* = 0.54, *P* < 0.005) [36]. Additional findings include bushy capillaries (*P* < 0.05) [32]. However, Marino *et al.* found no associations between scleroderma patterns and PAH [38]. The small number of cases (*n* = 5) may explain the null findings.

Nailfold patterns may also be used to identify patients at risk of developing PAH. In a cohort study of 40 patients with systemic sclerosis, Voilliot *et al* [23]. reported that advanced scleroderma patterns were an independent predictor of risk (adjusted hazard ratio 9.1 and 95% confidence interval (CI), 1.1–74.8) after a mean follow-up of 25 months. In addition, dilated capillary loops were present in all 10 patients with raised mPAP (>19 mmHg) [37].

Nailfold findings have also been reported in patients with chronic thromboembolic pulmonary hypertension (CTEPH). In the same study by Arvanitaki *et al* [31]., patients with CTEPH had reduced capillary density (8.0 vs. 9.7 loops/mm; *P* < 0.001) and increased capillary width (39.2 vs. 29.5 μm; *P* < 0.001). Patients also had nonspecific nailfold findings, which included thrombosis and ramifications.

### Systemic hypertension

Five studies investigated nailfold changes in 146 patients with systemic hypertension (Table 2). Capillary dimensions did not differ [26,30] but reduced red cell velocity was observed in patients with controlled [26] and uncontrolled [30] hypertension when compared with normotensive controls (0.99 vs. 1.14 mm/s; *P* < 0.05; 0.98 vs. 1.17 mm/s; *P* < 0.05). In two studies by Serné et al., patients with uncontrolled hypertension had reduced maximal [28] and perfused [27,28] capillary density, suggesting functional and structural loss of capillary density.

**Table 2:** Nailfold findings in patients with hypertension

First author	*N* a	Diagnostic criteria	BD	LD	RCV	Additional information
Junqueira *et al*., [30]	50 (25)	≥140/90 mmHg (uncontrolled) or <140/90 mmHg (controlled) and receiving treatment	**↔**	**↔**	**↓**	Reduced RCV in patients with uncontrolled hypertension
Cheng *et al*., [25]	24 (91)	≥130 mmHg and not receiving treatment	**↔**			Capillary density did not correlate with SBP or DBP
Penna *et al*., [26]	28 (19)	<140/90 mmHg and receiving treatment	**↓**	**↔**	**↓**	
Serné *et al*., [27]	18 (18)	>140/90 mmHg and previously receiving treatment	**↔**			Reduced perfused capillary density
Serné *et al*., [28]	26 (26)	>140/90 mmHg and not receiving treatment	**↔**			Reduced maximal and perfused capillary density

BD, baseline capillary density; LD, loop dimensions; N, sample size; RCV, red cell velocity.

a Sample size of patients with the disease of interest and the number of controls in brackets; ↑ increased; ↓ decreased; ↔ preserved.

Patients with controlled hypertension had reduced baseline capillary density [26], a finding not observed in patients with uncontrolled [25,27,28,30]. Patients with controlled hypertension received a formal diagnosis up to 10 years before the study [26], whereas patients with uncontrolled hypertension were diagnosed during the study [25] or had an unspecified disease duration [27,28,30].

### Vasculitis

Three types of vasculitis were assessed (Table 3). Patients with Takayasu arteritis had shorter capillaries (236 vs. 356 µm; *P* = 0.026) with reduced venous limb diameter (11.3 vs. 15.5 µm; *P* = 0.049) [29]. In addition, tortuous capillaries were a notable feature (*P* < 0.05). No difference in capillary density or other capillary dimensions was reported. Patients with Behçet’s syndrome had dilated capillaries and haemorrhages, but do not display other morphological features [39]. Children with Kawasaki disease had increased capillary dimensions, reduced red cell velocity and reduced capillary density [20].

**Table 3:** Nailfold findings in patients with vasculitis and other cardiovascular diseases

First author	*N*a	Condition	CD	LD	RCV	Abnormal morphology
Javinani *et al*., [29]	15 (15)	Takayasu arteritis	**↔**	**↓**		Tortuous
Aytekin *et al*., [39]	40 (40)	Behçet’s syndrome		**↑**		Haemorrhages
Huang *et al*., [20]	64 (36)	Kawasaki disease	**↓**	**↑**	**↓**	
Yüksel *et al*., [40]	58 (30)	Heart failure				Abnormal morphology in patients with heart failure with a preserved ejection fraction
Colaci *et al*., [41]	8 (125)	Aortic aneurysm				Scleroderma patterns
Voilliot *et al*., [24]	9 (41)	Cardiovascular eventsb				Scleroderma patterns

CD, capillary density; LD, loop dimensions; N, sample size; RCV, red cell velocity.

a Sample size of patients with the disease of interest and the number of controls in brackets.

b defined as cardiovascular mortality, hospitalisation due to heart failure, cardiac arrhythmias, pulmonary hypertension, stroke or peripheral arterial disease; ↑ increased; ↓ decreased; ↔ preserved.

### Other cardiovascular diseases

Abnormal morphology was more commonly observed among patients with heart failure with a preserved ejection fraction compared to heart failure with a reduced ejection fraction or healthy controls (*P* < 0.05) [40]. Among patients with systemic sclerosis, scleroderma nailfold patterns were used to detect the presence of an aortic aneurysm (*P* = 0.03) [41] or stratify the risk of cardiovascular events [24]. Advanced scleroderma pattern was an independent predictor of cardiovascular risk (*P* = 0.03) [24].

### Quality of evidence

The risk of bias was assessed using the Quality Assessment Tool for Observational Cohort and Cross-Sectional Studies (Supplementary Table S2, Supplemental Digital Content 1, http://links.lww.com/BPMJ/A182). Inter-rater agreement was 79% with a Cohen’s kappa of 0.70 (95% CI, 0.63–0.76), suggesting substantial agreement. No study fulfilled the entire criteria. The study by Huang *et al.* was given a poor rating because the siblings of patients were used as healthy controls [20], violating the assumption needed for the analysis of variance [42].

Investigators were blinded to the clinical history of the participant. Diagnostic measurements were repeated as appropriate. However, most studies did not exclude conditions associated with altered nailfold findings [23,24,41]. Only one study performed a power calculation [31]. As such, the studies with small sample sizes were unable to detect a statistically significant difference in nailfold findings. These studies tend to involve patients with rare diseases, for example, idiopathic PAH or Eisenmenger syndrome [31,35]. Furthermore, the use of echocardiography instead of right heart catheterisation in the control group may have left out subclinical cases of PAH [33,34].

## Discussion

This systematic review has identified published studies using NVC in patients with CVD. Given the extensive use of NVC in connective tissue disease, the evidence base is largely derived from diseases secondary to connective tissue disease, notably PAH.

Patients with PAH had reduced capillary density and increased capillary dimensions, a finding that was present regardless of the underlying cause (Table 1). Similar quantitative features were observed in patients with CTEPH, suggesting common underlying pathophysiology. In both conditions, mechanical stress [43] or a non-resolving pulmonary thrombus leads to capillary rarefaction and increased pulmonary vascular resistance [31]. The results of this systematic review support the hypothesis that changes in the peripheral microcirulation may be observed in patients with pulmonary vascular disease [31].

Patients with established but controlled hypertension had reduced baseline capillary density [26], which suggests that antihypertensive medications may not be effective in improving microvascular function. In addition, functional changes to the microcirculation precede structural changes, and capillary dimensions did not differ. Evidence supporting the use of NVC in other CVD is scarce and used NVC techniques with low magnification.

The findings of this review agree with previous studies. A meta-analysis of six observational studies involving patients with systemic sclerosis found a statistically nonsignificant reduction in capillary density (mean difference −1.0  loops/mm; 95% CI, −2.0 to 0.0) and a statistically significant increase in capillary width (mean difference +10.9 µm; 95% CI, 2.5–19.4) among patients with PAH [44]. In addition, capillary density was significantly reduced (mean difference −1.2 loops/mm; 95% CI, −2.3 to −0.1) when the analysis was limited to studies using right heart catheterisation.

Nailfold findings have been reported in other cardiometabolic disease. Tortuous and ramified capillaries are a feature of diabetes [45]. Patients with pre-eclampsia had increased capillary length and apical diameter with reduced maximal capillary density [46]. The results contrast with studies in patients with hypertension, in which capillary dimensions were preserved [26,30]. The differences reflect increases in capillary dimensions observed in normal pregnancy [47].

Capillaroscopy may be performed in the middle phalanges of the fingers. As the capillary loops are perpendicular to the skin surface, only capillary density can be measured. Patients with hypertension had reduced capillary density [48] that increases with blood pressure control [3,48].

### Possible mechanisms

**Fig. 3:**
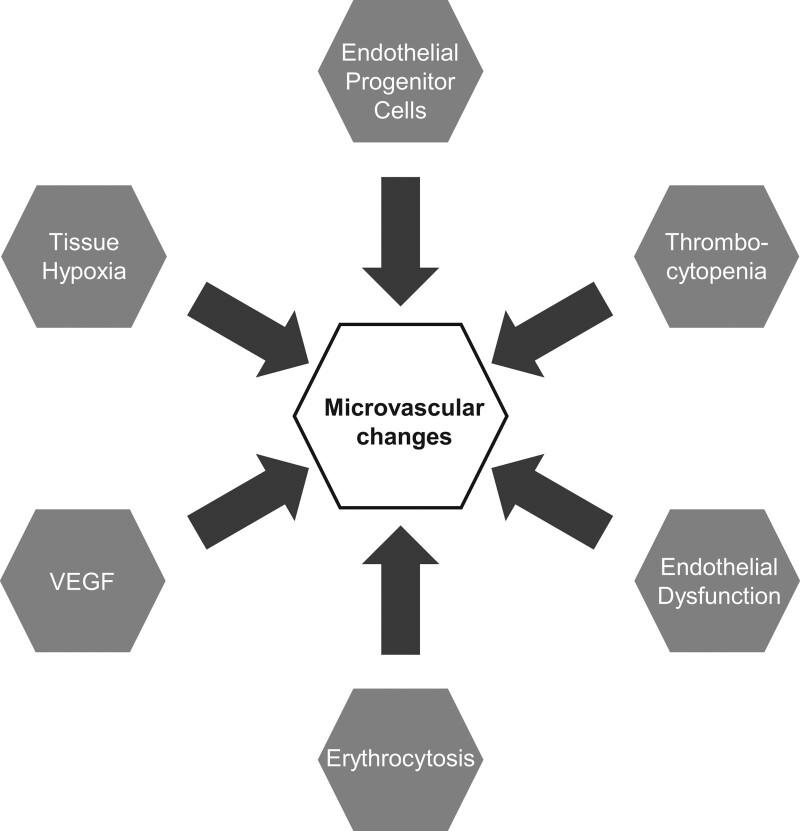
Possible mechanisms. Tissue hypoxia leads to vascular endothelial growth factor (VEGF) synthesis and erythrocytosis and defective angiogenesis. In patients with established cardiovascular disease, a decrease in circulating endothelial progenitor cells leads to reduced capillary density. Endothelial dysfunction and increased vasoconstrictor tone leads to decreased capillary recruitment and reduced red cell velocity. Thrombocytopenia may explain the presence of nailfold haemorrhages.

Several mechanisms have been proposed to explain the microvascular changes observed in CVD (Fig. 3). Tissue hypoxia in pulmonary hypertension [49,50] and vasculitis [29] increases vascular endothelial growth factor (VEGF) expression. The overexpression of VEGF promotes defective angiogenesis, which may lead to distorted capillaries and increased capillary dimensions [51]. The role of VEGF has been supported by patients with Eisenmenger syndrome reporting secondary erythrocytosis [52], an adaptive response to chronic hypoxaemia.

VEGF, along with cytokines interleukin-6 and tumour necrosis factor, leads to the mobilisation of endothelial progenitor cells (EPCs) [43]. In the later stages of the disease, a decrease in circulating EPCs leads to reduced capillary density, bleeding and thrombosis [53]. However, the presence of both proangiogenic and antiangiogenic VEGF isoforms further complicates the situation [51]. Additionally, thrombocytopenia may explain the presence of nailfold haemorrhages [54].

Given that the microcirculation, in particular the arterioles, are key in maintaining peripheral vascular resistance, the loss of capillaries and narrowing of existing capillaries may contribute to systemic and pulmonary hypertension [55]. Endothelial dysfunction and abnormal vasoconstrictor tone are a result of capillary rarefaction [25] and may contribute to the reductions in red cell velocity and capillary recruitment [25,26,56].

Tissue perfusion may be affected by the physiological properties of red blood cells, which include fluctuations in membrane thickness (membrane deformity) and aggregation [57]. Insults to the red blood cells may affect membrane deformity, notably glycation in patients with diabetes [57]. Functional changes have not been thoroughly studied in pulmonary hypertension, and we cannot rule out the possibility that these findings may be present in this population.

In addition, inflammation plays a role in endothelial dysfunction and the development of cardiovascular disease [30]. Elevated acute C-reactive proteins were observed in patients with arterial hypertension [30].

### Other forms of microvascular assessment

The parameters obtained from microvascular assessments have been illustrated (Supplementary Figure S1, Supplemental digital content, http://links.lww.com/BPMJ/A182). Laser-Doppler flowmetry measures blood flow in terms of red cell flux, which is the product of capillary density and red cell velocity [20]. Patients with Kawasaki disease had reduced red cell flux [20]. Conversely, no differences in baseline blood flow were reported in patients with hypertension [58]. In addition, patients with peripheral artery disease had lower resting feet temperature when measured with thermography [5].

### Strengths and limitations

This review provides an up-to-date summary on the use of NVC in patients with CVD. In addition to using standardised definitions of nailfold parameters, we illustrated the findings to avoid any ambiguity (Fig. 2). Furthermore, we explored how differences in CVD diagnosis and NVC measurement may have affected the results.

However, the term ‘microscopic angioscopy’ was introduced in the year 2000. As such, this review may have excluded earlier articles. The initial search identified 10 articles, whereas citation searching identified additional 12 additional articles. Older article did not specify the technique used and instead included the relevant parameters (e.g. capillary density) in the title and abstract [22,27,28,34,37].

### Gaps in knowledge

Limitations of NVC should be addressed. The reduced capillary visibility in patients with dark skin pigmentation [8] means that this patient population were excluded from these studies [26], which introduces elements of racial bias. Alteration to the spectrum of light may be considered. Alternatively, the other forms of microvascular assessment are unaffected by skin pigmentation.

During interpretation, nailfold images are compounded into a single mosaic, a process that is time-consuming and operator-dependent [59]. These limitations have led to the use of machine learning [59] and fast-track algorithms with excellent reliability [60]. However, the region of interest must be selected manually.

Abnormal nailfold findings are present in patients with comorbidities and receiving different medications [61], and may be present in participants without the systemic disease [62]. Large datasets of age- and gender-matched participants are required to define reference ranges. NVC findings should not be interpreted in isolation. A scoring system may facilitate this. In addition, the available evidence is derived from cross-sectional studies and does not allow the assessment of a temporal relationship. Well-powered, prospective cohort studies with repeated measurements are needed.

In conclusion, NVC has been used across the spectrum of CVD. This assessment may detect microvascular changes present in patients with CVD and predict cardiovascular risk in patients with systemic sclerosis. However, heterogeneity in NVC measurement, along with abnormal findings in healthy patients without systemic disease and other limitations outlined in this review, poses a significant barrier to the implementation of this inexpensive technique in the wider population.

## Acknowledgements

The authors are grateful for the nailfold images taken by the Clinical Research Facility, Queen Elizabeth University Hospital (Glasgow, UK).

M.W.S.L. has been supported by the James Patterson Bursary, D.S. by the European Commission’s Horizon 2020 Framework Programme (No 954798), and S.J.H.D., N.N.L., C.D. and P.J.C. by the British Heart Foundation Centre of Research Excellence Award (RE/18/6/34217).

### Conflicts of interest

There are no conflicts of interest.

### Data access

Data supporting this research has been presented in the paper. For any further requests please contact the corresponding author.

## Supplementary Material


