# Supplementary material for: Nailfold video-capillaroscopy in the study of cardiovascular disease: a systematic review
Source: Blood Press Monit. 2022 Oct 20;28(1):24–32. doi: 10.1097/MBP.0000000000000624 (PMC9815820; doi:10.1097/MBP.0000000000000624)
Supplement: Supplementary file 1 [file bpmj-28-24-s001.pdf]

Nailfold Video-Capillaroscopy in the Study of Cardiovascular Disease: A Systematic Review

Running head: Nailfold Findings in Cardiovascular Disease

Matthew W.S. Lim[1], Dellaneira Setjiadi[1], Stephen J.H. Dobbin[2], Ninian N. Lang[2], Christian Delles[1], and Paul J. Connelly[1]

[1]Institute of Cardiovascular and Medical Sciences, University of Glasgow, Glasgow, UK; [2]BHF Glasgow Cardiovascular Research Centre, University of Glasgow, Glasgow, UK.

Correspondence to Matthew W.S. Lim, Institute of Cardiovascular and Medical Sciences, University of Glasgow, 126 University Place, Glasgow G12 8TA, UK. e-mail: 2362717l@student.gla.ac.uk; Paul J. Connelly, University of Glasgow, Glasgow G12 8TA, UK. e-mail: paul.connelly@glasgow.ac.uk

Supplemental Digital Content

Table S1 | Reasons for exclusion

**Reason 1: Cardiovascular disease not studied**
1. Francischetti EA, Tibirica E, Da Silva EG, Rodrigues E, Celoria BM, De Abreu VG. Skin Capillary Density and Microvascular Reactivity in Obese Subjects with and without Metabolic Syndrome. *Microvasc Res* 2011; **81**:325-330.<br>2. Holm T, Aukrust P, Andreassen AK, Ueland T, Brosstad F, Frøland SS, et al. Peripheral endothelial dysfunction in heart transplant recipients: Possible role of proinflammatory cytokines. *Clin Transplant* 2000; **14**:218-225.<br>3. Obert P, Walther G, Dutheil F, Lesourd B, Chapier R, Courteix D, et al. Regional myocardial function abnormalities are associated with macro- and microcirculation dysfunction in the metabolic syndrome: the RESOLVE study. *Heart Vessels* 2018; **33**:688-694.<br>4. Strain WD, Chaturvedi N, Bulpitt CJ, Rajkumar C, Shore AC. Albumin excretion rate and cardiovascular risk: Could the association be explained by early microvascular dysfunction? *Diabetes* 2005; **54**:1816-1822.<br>5. Walther G, Obert P, Dutheil F, Chapier R, Lesourd B, Naughton G, et al. Metabolic syndrome individuals with and without type 2 diabetes mellitus present generalized vascular dysfunction: Cross-sectional study. *Arterioscler Thromb Vasc Biol* 2015; **35**:1022-1029.
**Reason 2: Lack of a comparator group**
1. Belcaro G, Ledda A, Hu S, Cesarone MR, Feragalli B, Dugall M. Grape seed procyanidins in pre- and mild hypertension: A registry study. *Evid Based Complement Alternat Med* 2013; **2013**.<br>2. van der Horst M, van Weissenbruch MM, de Vries JI. Thrombophilia mediates lowering cardiovascular risk factors in women with a history of preeclampsia. *Hypertens Pregnancy* 2011; **30**:421-432.

3. Widmer LW, Vikatmaa P, Aho P, Lepäntalo M, Venermo M. Reliability and repeatability of toe pressures measured with laser Doppler and portable and stationary photoplethysmography devices. *Ann Vasc Surg* 2012; **26**:404-410.

Reason 3: Use of an invasive test

1. Craighead DH, Alexander LM. Menthol-induced cutaneous vasodilation is preserved in essential hypertensive men and women. *Am J Hypertens* 2017; **30**:1156-1162.
2. Craighead DH, Wang H, Santhanam L, Alexander LM. Acute lysyl oxidase inhibition alters microvascular function in normotensive but not hypertensive men and women. *American Journal of Physiology - Heart and Circulatory Physiology* 2018; **314**:H424-H433.
3. Dillon GA, Greaney JL, Shank S, Leuenberger UA, Alexander LM. AHA/ACC-defined stage 1 hypertensive adults do not display cutaneous microvascular endothelial dysfunction. *American Journal of Physiology - Heart and Circulatory Physiology* 2020; **319** :H539-H546.
4. Greaney JL, Kutz JL, Shank SW, Jandu S, Santhanam L, Alexander LM. Impaired Hydrogen Sulfide-Mediated Vasodilation Contributes to Microvascular Endothelial Dysfunction in Hypertensive Adults. *Hypertension* 2017; **69**:902-909.
5. Stanhewicz AE, Alexander LM. Local angiotensin-(1–7) administration improves microvascular endothelial function in women who have had preeclampsia. *American Journal of Physiology - Regulatory Integrative and Comparative Physiology* 2020; **318**:R148-R155.
6. Stanhewicz AE, Dillon GA, Serviente C, Alexander LM. Acute systemic inhibition of inflammation augments endothelium-dependent dilation in

women with a history of preeclamptic pregnancy. *Pregnancy Hypertens* 2022; **27**:81-86.

7. Stanhewicz AE, Jandu S, Santhanam L, Alexander LM. Alterations in endothelin type B receptor contribute to microvascular dysfunction in women who have had preeclampsia. *Clin Sci* 2017; **131**:2777-2789.
8. Stanhewicz AE, Jandu S, Santhanam L, Alexander LM. Increased Angiotensin II Sensitivity Contributes to Microvascular Dysfunction in Women Who Have Had Preeclampsia. *Hypertension* 2017; **70**:382-389.
9. Stewart JM, Ocon AJ, Clarke D, Taneja I, Medow MS. Defects in cutaneous angiotensin-converting enzyme 2 and angiotensin-(1-7) production in postural tachycardia syndrome. *Hypertension* 2009; **53**:767-774.
10. Stewart JM, Ocon AJ, Medow MS. Ascorbate improves circulation in postural tachycardia syndrome. *American Journal of Physiology - Heart and Circulatory Physiology* 2011; **301**:H1033-H1042.
11. Stewart JM, Taneja I, Glover J, Medow MS. Angiotensin II type 1 receptor blockade corrects cutaneous nitric oxide deficit in postural tachycardia syndrome. *American Journal of Physiology - Heart and Circulatory Physiology* 2008; **294**:H466-H473.
12. Turner CG, Miller JT, Otis JS, Hayat MJ, Quyyumi AA, Wong BJ. Cutaneous sensory nerve-mediated microvascular vasodilation in normotensive and prehypertensive non-Hispanic Blacks and Whites. *Physiological Reports* 2020; **8**.
13. Wong BJ, Turner CG, Miller JT, Walker DC, Sebeh Y, Hayat MJ, et al. Sensory nerve-mediated and nitric oxide-dependent cutaneous vasodilation in normotensive and prehypertensive non-Hispanic blacks and whites.

American Journal of Physiology - Heart and Circulatory Physiology 2020; **319**:H271-H281.
**Reason 4: Written in foreign language**
1. Karpova IE, Fedorovich AA, Soboleva GN, Samoylenko LE, Rogoza AN, Karpov YA. Laser dopplerographic flowmetry in assessment of functional condition of the skin microvessels in patients with microvascular angina. *Russian Journal of Cardiology* 2015; **119**:58-63.
**Reason 5: Measurements not provided**
1. Cseprekál O, Egresits J, Tabák Á, Nemcsik J, Járai Z, Babos L, et al. The significance of micro- and macrovascular biomarkers on cardiovascular outcome in chronic kidney disease: a prospective cohort study. *J Hum Hypertens* 2016; **30**:449-455.
**Reason 6: Not relevant test used**
1. Aellen J, Dabiri A, Heim A, Liaudet L, Burnier M, Ruiz J, et al. Preserved Capillary Density of Dorsal Finger Skin in Treated Hypertensive Patients with or without Type 2 Diabetes. *Microcirculation* 2012; **19**:554-562.<br>2. Agarwal SC, Allen J, Murray A, Purcell IF. Laser Doppler assessment of dermal circulatory changes in people with coronary artery disease. *Microvasc Res* 2012; **84**:55-59.<br>3. Agra KF, Pontes IEA, da Silva JR, Jr., Figueiroa JN, Clough GF, Alves JGB. Impaired neurovascular reactivity in the microvasculature of pregnant women with preeclampsia. *Microcirculation* 2017; **24**.<br>4. Andersson SE, Edvinsson ML, Alving K, Edvinsson L. Vasodilator effect of endothelin in cutaneous microcirculation of heart failure patients. *Basic and Clinical Pharmacology and Toxicology* 2005; **97**:80-85.

5. Andreassen AK, Kirkebøen KA, Gullestad L, Svein S, Kvernebo K. Effect of heart transplantation on impaired peripheral microvascular perfusion and reactivity in congestive heart failure. *Int J Cardiol* 1998; **65**:33-40.
6. Barcelos A, Tibirica E, Lamas C. Evaluation of microvascular endothelial function and capillary density in patients with infective endocarditis using laser speckle contrast imaging and video-capillaroscopy. *Microvasc Res* 2018; **118**:61-68.
7. Barr LC, Pudwell J, Smith GN. Postpartum microvascular functional alterations following severe preeclampsia. *American Journal of Physiology - Heart and Circulatory Physiology* 2021; **320**:H1393-H1402
8. Blaauw J, Graaff R, van Pampus MG, van Doormaal JJ, Smit AJ, Rakhorst G, et al. Abnormal endothelium-dependent microvascular reactivity in recently preeclamptic women. *Obstet Gynecol* 2005; **105**:626-632.
9. Bondesson SM, Edvinsson ML, Pettersson T, Edvinsson L. Reduced peripheral vascular reactivity in refractory angina pectoris: Effect of enhanced external counterpulsation. *Journal of Geriatric Cardiology* 2011; **8**:215-223.
10. Borges JP, Lopes GO, Verri V, Coelho MP, Nascimento PMC, Kopiler DA, et al. A novel effective method for the assessment of microvascular function in male patients with coronary artery disease: A pilot study using laser speckle contrast imaging. *Braz J Med Biol Res* 2016; **49**.
11. Brown RA, Lip GYH, Varma C, Shantsila E. Impact of Mon2 monocyte-platelet aggregates on human coronary artery disease. *Eur J Clin Invest* 2018; **48**.
12. Butt M, Khair OA, Dwivedi G, Shantsila A, Shantsila E, Lip GYH. Myocardial perfusion by myocardial contrast echocardiography and endothelial dysfunction in obstructive sleep apnea. *Hypertension* 2011; **58**:417-424.

13. Casanova F, Adingupu DD, Adams F, Gooding KM, Looker HC, Aizawa K, et al. The impact of cardiovascular co-morbidities and duration of diabetes on the association between microvascular function and glycaemic control. *Cardiovasc Diabetol* 2017; **16**:114.
14. Caspary L, Nordbruch S, Lange R, Creutzig A. Circadian variation of skin perfusion in arterial occlusive disease. *Vasa - Journal of Vascular Diseases* 1997; **26**:194-198.
15. Cui J, Arbab-Zadeh A, Prasad A, Durand S, Levine BD, Crandall CG. Effects of heat stress on thermoregulatory responses in congestive heart failure patients. *Circulation* 2005; **112**:2286-2292.
16. Farkas K, Fábián E, Kolossváry E, Járai Z, Farsang C. Noninvasive assessment of endothelial dysfunction in essential hypertension: Comparison of the forearm microvascular reactivity with flow-mediated dilatation of the brachial artery. *Int J Angiol* 2003; **12**:224-228.
17. Farkas K, Kolossváry E, Járai Z, Nemcsik J, Farsang C. Non-invasive assessment of microvascular endothelial function by laser doppler flowmetry in patients with essential hypertension. *Atherosclerosis* 2004; **173**:97-102.
18. Farkas K, Nemcsik J, Kolossváry E, Járai Z, Nádory E, Farsang C, et al. Impairment of skin microvascular reactivity in hypertension and uraemia. *Nephrol Dial Transplant* 2005; **20**:1821-1827.
19. Fronek A, Allison M. Noninvasive evaluation of endothelial activity in healthy and diseased individuals. *Vasc Endovascular Surg* 2014; **48**:134-138.
20. Glazkova PA, Kulikov DA, Glazkov AA, Terpigorev SA, Rogatkin DA, Shekhyan GG, et al. Reactivity of skin microcirculation as a biomarker of cardiovascular events. Pilot study. *Clin Hemorheol Microcirc* 2021; **78**:247-257.

21. Hellmann M, Tarnawska M, Dudziak M, Dorniak K, Roustit M, Cracowski JL. Reproducibility of flow mediated skin fluorescence to assess microvascular function. *Microvasc Res* 2017; **113**:60-64.
22. Holm T, Aukrust P, Andreassen AK, Ueland T, Brosstad F, Frøland SS, et al. Peripheral endothelial dysfunction in heart transplant recipients: Possible role of proinflammatory cytokines. *Clin Transplant* 2000; **14**:218-225.
23. Ilo A, Romsi P, Mäkelä J. Infrared Thermography as a Diagnostic Tool for Peripheral Artery Disease. *Advances in Skin and Wound Care* 2020; **33**:482-488.
24. Ishii T, Takabe S, Yanagawa Y, Ohshima Y, Kagawa Y, Shibata A, et al. Laser Doppler blood flowmeter as a useful instrument for the early detection of lower extremity peripheral arterial disease in hemodialysis patients: An observational study. *BMC Nephrol* 2019; **20**.
25. Jaap AJ, Shore AC, Tooke JE. The Influence of Hypertension on Microvascular Blood Flow and Resistance to Flow in the Skin of Patients with Type 2 (Non-insulin-dependent) Diabetes. *Diabet Med* 1994; **11**:883-887.
26. Jadhav ST, Ferrell WR, Petrie JR, Scherbakova O, Greer IA, Cobbe SM, et al. Microvascular Function, Metabolic Syndrome, and Novel Risk Factor Status in Women With Cardiac Syndrome X. *Am J Cardiol* 2006; **97**:1727-1731.
27. Kaiser SE, Sanjuliani AF, Estato V, Gomes MB, Tibiriçá E. Antihypertensive Treatment Improves Microvascular Rarefaction and Reactivity in Low-Risk Hypertensive Individuals. *Microcirculation* 2013; **20**:703-716.
28. Kurio GH, Zhiroff KA, Jih LJ, Fronek AS, Burns JC. Noninvasive determination of endothelial cell function in the microcirculation in Kawasaki syndrome. *Pediatr Cardiol* 2008; **29**:121-125.

29. Monostori P, Baráth Á, Fazekas I, Hódi E, Máté A, Farkas I, et al. Microvascular reactivity in lean, overweight, and obese hypertensive adolescents. *Eur J Pediatr* 2010; **169**:1369-1374.
30. Murphy MSQ, Vignarajah M, Smith GN. Increased microvascular vasodilation and cardiovascular risk following a pre-eclamptic pregnancy. *Physiological Reports* 2014; **2**.
31. Nazzaro P, Schirosi G, Mezzapesa D, Petruzzellis M, Pascazio L, Serio G, et al. Effect of clustering of metabolic syndrome factors on capillary and cerebrovascular impairment. *Eur J Intern Med* 2013; **24**:183-188.
32. Östlund Papadogeorgos N, Jörneskog G, Bengtsson M, Kahan T, Kalani M. Severely impaired microvascular reactivity in diabetic patients with an acute coronary syndrome. *Cardiovasc Diabetol* 2016; **15**.
33. Parshakov A, Zubareva N, Podtaev S, Frick P. Detection of Endothelial Dysfunction Using Skin Temperature Oscillations Analysis During Local Heating in Patients With Peripheral Arterial Disease. *Microcirculation* 2016; **23**:406-415.
34. Prázný M, Ježková J, Horová E, Lazárová V, Hána V, Kvasnička J, et al. Impaired microvascular reactivity and endothelial function in patients with Cushing's syndrome: Influence of arterial hypertension. *Physiol Res* 2008; **57**:13-22.
35. Ramsay JE, Ferrell WR, Crawford L, Wallace AM, Greer IA, Sattar N. Divergent metabolic and vascular phenotypes in pre-eclampsia and intrauterine growth restriction: Relevance of adiposity. *J Hypertens* 2004; **22**:2177-2183.
36. Sanchez-Garcia ME, Ramirez-Lara I, Gomez-Delgado F, Yubero-Serrano EM, Leon-Acuña A, Marin C, et al. Quantitative evaluation of capillaroscopic

microvascular changes in patients with established coronary heart disease. *Med Clin (Barc)* 2018; **150**:131-137.

37. Shantsila A, Dwivedi G, Shantsila E, Butt M, Beevers DG, Lip GYH. Persistent macrovascular and microvascular dysfunction in patients with malignant hypertension. *Hypertension* 2011; **57**:490-496.
38. Shantsila E, Wrigley B, Shantsila A, Tapp LD, Blann AD, Gill PS, et al. Ethnic differences in macrovascular and microvascular function in systolic heart failure. *Circ Heart Fail* 2011; **4**:754-762.
39. Shisen J, Gorai Y, xu SZ. Development of a New Laser-Doppler Microvelocimetering and Its Application in Patients with Coronary Artery Stenosis. *Angiology* 1994; **45**:225-230.
40. Siniewicz K, Wiecek B, Baszczynski J, Zwolenik S. Thermal imaging before and after physical exercises in children with orthostatic disorders of the cardiovascular system. *Thermology International* 2002; **12**:139-146.
41. Souza EG, De Lorenzo A, Huguenin G, Oliveira GMM, Tibiriçá E. Impairment of systemic microvascular endothelial and smooth muscle function in individuals with early-onset coronary artery disease: Studies with laser speckle contrast imaging. *Coron Artery Dis* 2014; **25**:23-28.
42. Spaan JJ, Houben AJ, Musella A, Ekhart T, Spaanderman ME, Peeters LL. Insulin resistance relates to microvascular reactivity 23 years after preeclampsia. *Microvasc Res* 2010; **80**:417-421.
43. Stewart J, Kohen A, Brouder D, Rahim F, Adler S, Garrick R, et al. Noninvasive interrogation of microvasculature for signs of endothelial dysfunction in patients with chronic renal failure. *American Journal of Physiology - Heart and Circulatory Physiology* 2004; **287**:H2687-H2696.

44. Stewart JM, Ocon AJ, Clarke D, Taneja I, Medow MS. Defects in cutaneous angiotensin-converting enzyme 2 and angiotensin-(1-7) production in postural tachycardia syndrome. *Hypertension* 2009; **53**:767-774.
45. Stewart JM, Ocon AJ, Medow MS. Ascorbate improves circulation in postural tachycardia syndrome. *American Journal of Physiology - Heart and Circulatory Physiology* 2011; **301**:H1033-H1042.
46. Stiefel P, Moreno-Luna R, Vallejo-Vaz AJ, Beltrán LM, Costa A, Gómez L, et al. Which parameter is better to define endothelial dysfunction in a test of postocclusive hyperemia measured by Laser-Doppler flowmetry? *Coron Artery Dis* 2012; **23**:57-61.
47. Strain WD, Hughes AD, Mayet J, Wright AR, Kooner J, Chaturvedi N, et al. Attenuation of microvascular function in those with cardiovascular disease is similar in patients of Indian Asian and European descent. *BMC Cardiovasc Disord* 2010; **10**:3.
48. Strain WD, Hughes AD, Mayet J, Wright AR, Kooner J, Chaturvedi N, et al. Attenuated systemic microvascular function in men with coronary artery disease is associated with angina but not explained by atherosclerosis. *Microcirculation* 2013; **20**:670-677.
49. Tibirica E, Souza EG, De Lorenzo A, Oliveira GMM. Reduced systemic microvascular density and reactivity in individuals with early onset coronary artery disease. *Microvasc Res* 2015; **97**:105-108.
50. Verri V, Brandão AA, Tibirica E. Penile microvascular endothelial function in hypertensive patients: Effects of acute type 5 phosphodiesterase inhibition. *Braz J Med Biol Res* 2018; **51**.
51. Zhao J, Li N, Wang J, Huang Z, Zhang Y, He Z, et al. Cutaneous microvascular function in patients with obstructive or non-obstructive

coronary artery disease evaluated by laser speckle contrast imaging.

Microvasc Res 2019; **125**.

Fig. S1 | Assessment of microvascular structure and function

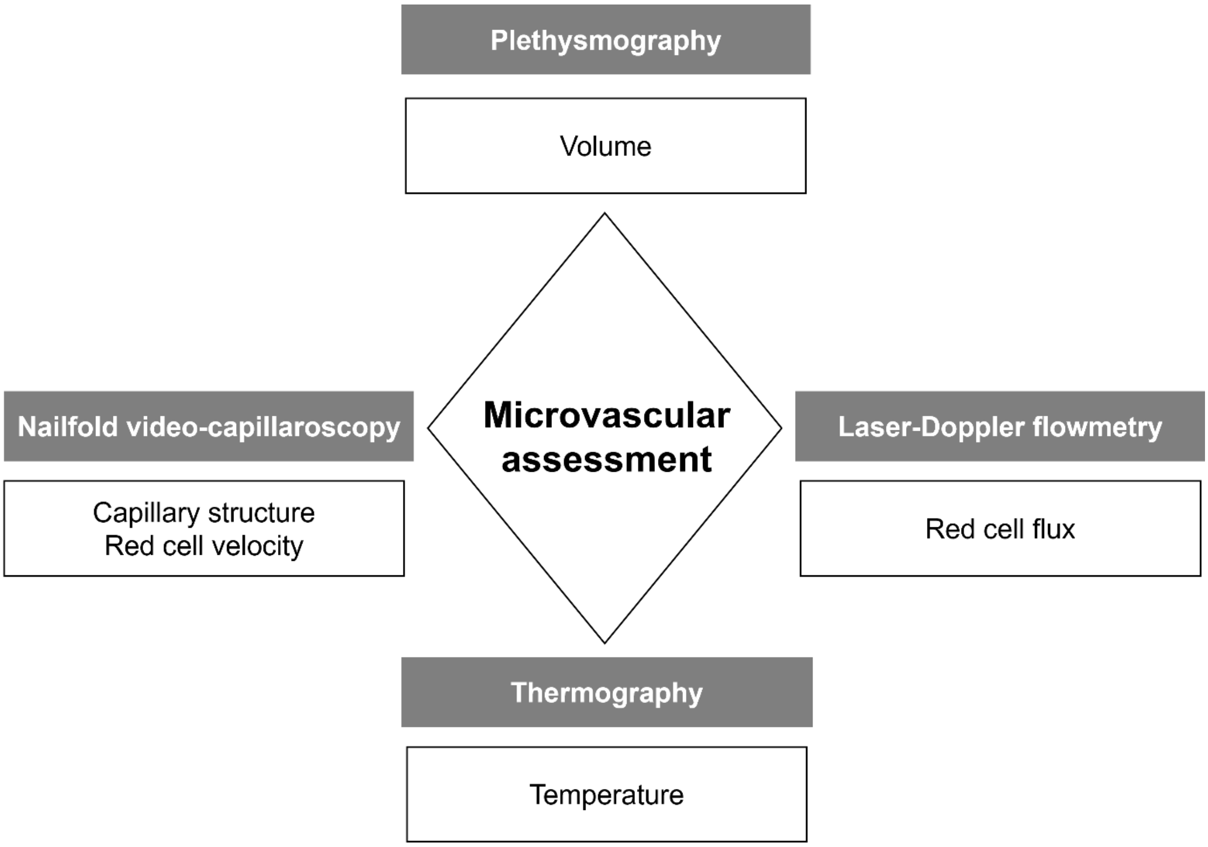

Available microvascular assessments include nailfold video-capillaroscopy (NVC), venous occlusion plethysmography, laser-Doppler flowmetry and thermography. Of the four methods, only NVC assesses microvascular structure. Blood flow may be measured as red cell velocity (NVC), changes in forearm volume (plethysmography), red cell flux (laser-Doppler flowmetry) or changes in temperature (thermography). Red cell flux is the product of capillary density and red cell velocity.

Table S2 | Quality assessment

Reference	Q1	Q2	Q3	Q4	Q5	Q6	Q7	Q8	Q9	Q10	Q11	Q12	Q13	Q14	QR
Yüksel [40]	Y	Y	NR	Y	N	N	N	Y	N	N	N	N	NA	Y	Fair
Colaci [41]	Y	Y	Y	Y	N	N	N	NA	Y	N	N	N	NA	N	Fair
Arvanitaki [21]	Y	Y	NR	Y	N	N	N	Y	N	N	Y	Y	NA	Y	Good
Arvanitaki [31]	Y	Y	NR	Y	Y	N	N	N	Y	N	Y	Y	NA	Y	Good
Arvanitaki [35]	Y	Y	NR	Y	N	N	N	NA	Y	N	Y	Y	NA	Y	Good
Javinani [29]	Y	Y	NR	Y	N	N	N	NA	Y	N	Y	Y	NA	Y	Good
Junqueira [30]	Y	Y	NR	Y	N	N	N	Y	Y	Y	Y	N	NA	Y	Fair
Corrado [32]	Y	N	NR	Y	N	N	N	N	Y	N	Y	N	NA	N	Fair
Voilliot [23]	Y	Y	Y	Y	N	N	Y	N	Y	N	Y	NA	Y	N	Fair
Voilliot [24]	Y	Y	Y	Y	N	N	Y	N	Y	N	Y	NA	Y	N	Fair
Aytekin [39]	Y	Y	NR	Y	N	N	N	NA	Y	N	Y	Y	NA	N	Fair
Riccieri [36]	Y	Y	NR	Y	N	N	N	Y	Y	N	Y	N	NA	N	Fair
Marino [38]	Y	Y	NR	Y	N	N	N	N	Y	N	Y	Y	NA	N	Fair
Huang [20]	Y	Y	NR	Y	N	N	N	NA	Y	N	N	N	NA	Y	Poor
Cheng [25]	Y	Y	N	Y	N	N	N	Y	Y	Y	Y	Y	NA	Y	Good
Hofstee [33]	Y	Y	NR	N	N	Y	N	Y	N	N	Y	Y	Y	N	Fair

Penna [26]	Y	Y	NR	Y	N	N	N	N	Y	Y	Y	Y	NA	Y	Fair
Serné [27]	Y	N	NR	Y	N	N	N	Y	Y	Y	Y	Y	NA	Y	Good
Serné [28]	Y	N	NR	Y	N	N	N	N	Y	Y	Y	Y	NA	Y	Fair
Greidinger [34]	Y	Y	NR	Y	N	N	N	N	Y	N	Y	Y	NA	N	Fair
Ong [22]	Y	Y	NR	Y	N	N	N	N	Y	N	Y	N	NA	N	Fair
Ohtsuka [37]	Y	Y	NR	Y	N	N	N	N	Y	N	Y	N	NA	N	Fair

Abbreviations: QR- Quality rating; Y - Yes; N - No; NR - Not reported; NA - Not applicable
